# Randomized Controlled Trial on the Effects of a Combined Intervention of Computerized Cognitive Training Preceded by Physical Exercise for Improving Frailty Status and Cognitive Function in Older Adults

**DOI:** 10.3390/ijerph18041396

**Published:** 2021-02-03

**Authors:** Ruby Yu, Grace Leung, Jean Woo

**Affiliations:** 1 Department of Medicine and Therapeutics, Faculty of Medicine, The Chinese University of Hong Kong, Hong Kong, China; jeanwoowong@cuhk.edu.hk; 2 CUHK Jockey Club Institute of Ageing, The Chinese University of Hong Kong, Hong Kong, China; gracesmleung@cuhk.edu.hk

**Keywords:** cognitive function, computerized cognitive training, executive functions, frailty, verbal memory

## Abstract

(1) Objective: This study examined the effects of a combined intervention of *Brainastic* computerized cognitive training (CCT) preceded by physical exercise (PE) for improving frailty status and cognitive function in older adults. (2) Methods: Older adults aged 50 years or older attending elderly centers, without frailty/history of cognitive impairment, were randomly allocated into either a 12-week (i) multi-domain CCT + PE (*n* = 117), (ii) two-domain CCT + PE (*n* = 116) or (iii) video watching + PE (i.e., control, *n* = 114). *Brainastic* is an online application for cognitive training through video games. The multi-domain CCT targeted memory, attention, executive function, flexibility and visuospatial ability while the two-domain CCT targeted memory and attention. PE included both aerobic and resistance exercises. Outcomes were changes in frailty levels as measured with a simple frailty questionnaire (FRAIL), global cognition as measured with the Rapid Cognitive Screen (RCS), total learning and verbal memory abilities as measured with the Hong Kong List Learning Test (HKLLT), and executive functions as measured with the Frontal Assessment Battery (FAB) over 12 weeks. (3) Results: Participants in the intervention groups (multi-/two-domain CCT + PE) showed greater improvements in frailty status, total learning ability and verbal memory ability than control participants (all *p* < 0.05). The multi-domain CCT did not outperform the two-domain CCT in improving frailty status or cognitive function. The training effects were independent of the baseline cognition of the participants. (4) Conclusions: A combined intervention of multi-/two-domain CCT preceded by PE seemed to convey benefit over video watching preceded by PE in improving frailty status and cognitive function among older adults attending elderly centers.

## 1. Introduction

Functional and cognitive decline are associated with many adverse outcomes, which affect the quality of life of older adults [1,2]. Cognitive dysfunction or worsening of cognitive function may also be a precursor of mild neurodegenerative disorder (Mild Cognitive Impairment, MCI) and subsequently major neurodegenerative disorder (Dementia) [3,4]. Therefore, enhancing functional ability and delaying cognitive decline are important public health and social issues [5,6].

Many possible strategies to preserve or enhance functional ability and cognitive function in older adults have been investigated. Among these, the effects of physical exercise (PE) on reducing the risk of cognitive decline among adults with normal cognition are comparatively robust and promising [7]. The findings from meta-analyses of PE with regard to enhancing physical [8] and cognitive function [9,10,11] are also positive, whereas the improvements in cognition are considered specific to increased activation in regions of the prefrontal and parietal cortex, which translates into better cognitive performance [12]. Research on cognitive training that offers teaching mnemonic strategies or guided practice in specific standardized tasks designed to enhance particular cognitive functions such as memory, attention, fine-motor coordination, visual and auditory processing, are also linked to cognitive gains [13,14]. However, the potential effects of cognitive training on physical function are less studied. It has also been argued that cognitive training was mostly administered face to face with a trained professional, which is unlikely to be cost-effective or feasible for large-scale implementation.

With the penetration of new technologies, computerized cognitive training (CCT) has started to play a significant role in this field. Earlier studies have shown that CCT can improve memory [15], reaction time [16,17], speed and reasoning [18], learning and interference tendency [19] and verbal fluency [20] in older adults. Compared to traditional cognitive training, CCT simplifies the setting of the level of intensity in response to training progression. It also has the advantage of ease of scalability and community implementation. Some studies have suggested that CCT is associated with higher levels of satisfaction when compared with traditional cognitive training [21,22]. However, the effectiveness of CCT for improving cognitive function of older adults is still unclear, as the findings from other trials have been inconsistent [23]. Although some recent meta-analyses of randomized controlled trials (RCTs) indicated small-to-moderate beneficial effects of CCT on global cognition and some specific cognitive domains such as memory in healthy older adults [24] and adults with MCI [25,26], no differences were observed in relation to executive functions [24,25,26].

Previous studies have also found that multicomponent interventions combining physical, cognitive and/or social activities can bring greater cognitive benefits than single-component interventions [27,28], probably due to PE, especially aerobic exercise, and cognitive activities stimulating neurogenesis through independent but complementary pathways, such that combining these two types of activity could increase the number of neurons, improve learning and memory, and hence cognitive function [29,30]. However, there is less research on the combined effects of CCT and PE, although a few trials have reported beneficial effects on cognitive function in healthy older adults [31,32,33]. It also remains unclear to what extent such effects are of clinical value (e.g., experiencing reduced functional ability or reduction in the risk of dementia). Further RCTs are needed to examine the role of CCT to prevent functional and cognitive decline. Such studies may lead to the development of interventions for addressing loss of functional ability and cognitive decline in older adults.

In this study, we examined the combined effects of a 12-week CCT intervention preceded by PE, compared with an active control with video watching immediately followed by PE, on frailty, global cognition, total learning ability, verbal memory ability and executive functions in community-dwelling older adults. We also compared the training effects of a multi-domain CCT with a two-domain CCT and compared the effects of the interventions according to the baseline cognition of participants.

## 2. Materials and Methods 

### 2.1. Study Design

This is a 12-week RCT conducted from July 2018 to April 2019 comparing a CCT intervention using touch-screen video game technology preceded by PE and an active control among community-dwelling older adults in Hong Kong. Treatment outcomes were assessed at baseline (pre-), and post-treatment (i.e., 12 weeks). The study was carried out in four elderly centers of three Non-Government Organizations (NGOs) including the Jockey Club CADENZA Hub, St. James’ Settlement and the Hong Kong Society for Rehabilitation. The study was registered with the Australian New Zealand Clinical Trials Registry (Trial I.D.: ACTRN12618000993291).

### 2.2. Participants

**Figure 1:**
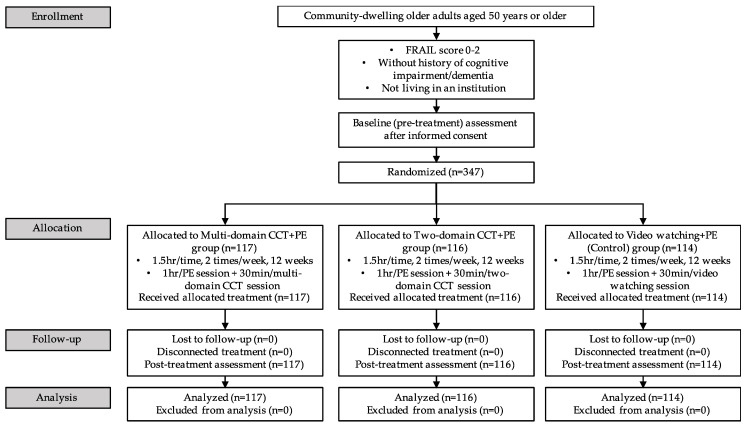
Flow chart of the study design. CCT = computerized cognitive training; FRAIL = the simple frailty questionnaire; PE = physical exercise.

Participants were recruited from communities in Hong Kong through a recruitment notice. Participants who responded to the notice were assessed for eligibility before being included in the study. Inclusion criteria were 50 years or older; robust or pre-frail (met specified cut-offs for 0–2 of the 5-item simple frailty questionnaire, FRAIL); without history of cognitive impairment/dementia, and not living in an institution. Exclusion criteria were severe medical conditions limiting the ability to complete the treatment; major neurological or psychiatric illness history; acetylcholinesterase inhibitor use; current substance abuse; significant communicative impairments; and concurrent enrolment in other cognitive studies, experimental therapies, or blinded treatments. All participants gave their informed consent for inclusion before they participated in the study. The study design is detailed in Figure 1.

### 2.3. Baseline Assessment and Randomization

After obtaining informed consent, a baseline (pre-) assessment was performed. Participants were randomly assigned into three groups: the multi-domain CCT + PE group (*n* = 117), the two-domain CCT + PE group (*n* = 116) and the video watching + PE group (control, *n* = 114). Block randomization was undertaken by randomizing participants within blocks such that an equal number of participants was assigned to each group in order to achieve equal samples sizes within each treatment group during the study period. The group allocation schedule was generated and managed by an investigator independent of participant recruitment and assessment.

### 2.4. Intervention

#### 2.4.1. The Multi-Domain CCT + PE Group

Participants randomized into the multi-domain CCT + PE visited the centers two times per week for 1.5 h (for 12 weeks), and performed a 1-h PE program (aerobic + resistance exercise) immediately followed by a 30-min session of *Brainastic* CCT targeting five cognitive domains (memory, attention, executive functions, flexibility and visuospatial ability) through 15 video games (game 1–3, 5–7 and 9–17). The *Brainastic* CCT was developed by Mindvivid Limited (Hong Kong Science Park, Hong Kong) and is performed on a tablet with each game targeting one of the five domains. Please refer to Table 1 for details of games. The PE program included an aerobic circuit training including 15 exercises such as marching, squatting and swinging arms and resistance training including 8–10 exercises, such as knee extension, chest stretching and torso stretching. TheraBands were used for the resistance training. The intensity of both the aerobic and resistance exercises increased throughout the program.

During each session, participants had access to only one prescribed track of games related to the same cognitive domain. The difficulty of each game gradually increased according to each participant’s performance from the previous session. A facilitator (trained research assistant) was available to provide instructions before each session and assist participants who had trouble accessing the training.

#### 2.4.2. The Two-Domain CCT + PE Group

Similar to the multi-domain CCT + PE group, participants randomized into the two-domain CCT + PE group visited the centers two times per week for 1.5 h (for 12 weeks), and performed aa 1-h PE program (aerobic + resistance exercise) immediately followed by a 30-min session of *Brainastic* CCT targeted memory and attention through eight video games (game 1-8). The games were also performed on a tablet. Please refer to Table 1 for details of games.

#### 2.4.3. Video Watching + PE (Control) Group

Participants randomized into the control group also visited the centers two times per week for 1.5 h (for 12 weeks), and performed a 1-h PE program (aerobic + resistance exercise) immediately followed by a 30-min session of video watching. There were 15 videos covering different topics on history, art, literature and science. Quizzes with multiple choice questions related to the video were undertaken afterwards to ensure active participation and learning during the session.

### 2.5. Outcome Measures

Measurement of outcomes took place at pre- and post-assessments (i.e., 12 weeks) by trained research assistants who were blinded to the participants’ group allocation, and independent of the interventional researchers who administered treatment.

#### 2.5.1. Frailty

The FRAIL scale is a simple frailty questionnaire to assess frailty status [34]. It includes five components: fatigue, resistance, ambulation, illnesses, and loss of weight. FRAIL scores range from 0–5 (i.e., 1 point for each component) with 0 representing robust, 1–2 pre-frail, and 3–5 frail status. The FRAIL scale has been validated for populations of African American patients [34], Chinese [35], and other Asian populations [36] and has been shown to predict disability and mortality [35,37].

#### 2.5.2. Cognitive Function

The Rapid Cognitive Screen (RCS) is a brief screening tool for cognitive dysfunction [38]. It includes three items from the Veteran Affairs Saint Louis University Mental Status (SLUMS) exam: memory in recalling four words (5 points), a clock drawing test (4 points; 2 points for hour markers, 2 points for time), and the ability to remember a story and the fact that Tung Chung is in the Islands District of Hong Kong (1 point). RCS scores range from 0 (the worst) to 10 (the best), with 0–5 representing at risk of dementia, 6–7 representing at risk of MCI, and 8–10 representing normal cognition.

The Hong Kong List Learning Test (HKLLT) is a Chinese verbal learning test, which emphasizes the evaluation of the processes and organization strategies involved in learning verbal information [39,40,41]. It consists of two 16-word lists with the words being two-character nouns. The words in the first list come from four categories and are randomly organized, while the second list consists of words from another four categories that are semantically clustered. In this study, participants were required to learn the first list through three learning trials and to recall as many words as possible after a 10-min delay to reflect verbal memory ability. The total number of correctly recalled words during the three learning trials gave the Total Learning (HKLLT-TL) score, which ranges from 0 (the worst) to 48 (the best). The total number of recalled words after the 10-min delay gave the Delay Recall Trial (HKLLT-DRT) score, which ranges from 0 (the worst) to 16 (the best).

The Frontal Assessment Battery (FAB) is a brief battery of neuropsychological tasks designed to assess frontal lobe function and screen for frontotemporal dementia [42]. It comprises six subtests: (1) conceptualization and abstract reasoning (similarities test), (2) mental flexibility (verbal fluency test), (3) motor programming and executive control of action (Lucia motor sequences), (4) resistance to interference (conflicting instructions), (5) inhibitory control (go-no-go test) and (6) environmental autonomy (prehension behavior). FAB scores range from 0–3 for each subtest and the total score is the sum of the six subtests ranging from 0 (the worse) to 18 (the best) (cut-off at 12, sensitivity of 77% and specificity of 87%) [43].

#### 2.5.3. Covariates

Demographic (age, sex, marital status, educational level and living arrangement) data was collected using a standardized questionnaire.

### 2.6. Statistical Analysis

Comparisons across treatment groups were performed by *t* tests for continuous variables or chi-squared (χ^2^) for categorical variables. The effects of intervention over 12 weeks on outcome measures were investigated based on the modified intention-to-treat (mITT) analysis, including all randomly assigned participants with complete baseline and week 12 follow-up assessments. The change in outcome measures from baseline to week 12 were performed by paired *t* tests. The mean differences of outcome measures between the intervention and the control groups were examined using ANCOVA, with adjustment for significant variable(s) assessed in the baseline characteristics comparisons (i.e., FAB score at baseline). The analyses were repeated according to the baseline values of the RCS score of the participants. All analyses were carried out using the Windows-based SPSS statistical Package (v. 26.0; IBM Corp, Armonk, NY) and *p* values of less than 0.05 were considered statistically significant.

## 3. Results

A total of 347 older adults aged 50 or older were randomly assigned into the multi-domain CCT + PE group (*n* = 117), the two-domain CCT + PE group (*n* = 116) and the control group (*n* = 114). The characteristics of the participants at baseline within each group are presented in Table 2. The mean age of the participants was 64.2 years, 85.6% were women and 68% were classified as pre-frail (data not shown). Comparison of the baseline characteristics did not show any statistical differences in age, sex, marital status, educational level, living arrangement, frailty levels and cognitive functions (RCS score, HKLLT-LT score, HKLLT-DRT score) between the groups, although FAB scores showed a difference (multi-domain CCT + PE, 14.5 ± 2.4, vs. two-domain CCT + PE, 15.6 ± 2.1, vs. control group, 14.7 ± 2.6, *p <* 0.01).

At week 12, FRAIL scores significantly decreased in the multi- and two-domain CCT + PE groups (multi-domain CCT + PE, −0.4 ± 1.0, *p* < 0.001; two-domain CCT + PE, −0.4 ± 0.8, *p* < 0.001) but not in the control group. The differences between the groups were significant (*p* < 0.05). Improvement in RCS scores were observed in both multi- and two-domain CCT + PE groups (multi-domain CCT + PE, 0.6 ± 2.0, *p* < 0.01; two-domain CCT + PE, 0.5 ± 1.8, *p* < 0.01), but not in the control group. Both HKTLL-TL and HKLLT-DRT scores increased in all study groups (all *p* < 0.01), but the increase was higher in the CCT + PE groups than in the control group (*p* < 0.05). FAB scores also increased in all study groups (all *p* < 0.001), but the differences between the groups were not significant. (Table 3).

Sub-group analyses were performed to examine the training effects according to the baseline RCS values of the participants. Among those with RCS ≥ 8 (*n* = 135), FRAIL score significantly decreased in both multi- and two-domain CCT + PE groups (multi-domain CCT + PE, −0.4 ± 1.0, *p* < 0.05; two-domain CCT + PE −0.4 ± 0.8, *p* < 0.01) at week 12, but not in the control group. Improvement in HKLLT-DRT scores were observed at week 12 in both multi- and two-domain CCT + PE groups (all *p* < 0.05), but not in the control group. The differences between the groups were also significant (*p* < 0.01). Scores of HKLLT-TL and FAB improved in all study groups (all *p* < 0.05), but the differences between the groups were not significant. Among those with RCS < 8 (*n* = 212), reduction in FRAIL scores were observed at week 12 in both multi- and two-domain CCT + PE groups (all *p* < 0.001), but not in the control group. The between group difference was significant (*p* < 0.05). Scores of HKLLT-TL, HKLLT-DRT and FAB improved in all study groups (all *p* < 0.01), but the differences between the groups were not significant (Table 4).

## 4. Discussion

In this study, we found that a 12-week combined intervention of CCT preceded by PE (aerobic + resistance exercise) would yield significantly larger benefits on frailty status and cognitive function than the control participants, who received PE followed by video watching which covered different topics on history, art, literature and science. However, the multi-domain CCT did not outperform the two-domain CCT in improving frailty status and cognitive function.

Few RCTs have evaluated the effects of CCT or the combined effects of CCT and PE on frailty status. In this respect, our findings indicate a significant decrease in the FRAIL score after the CCT interventions which were preceded by PE, but when video watching immediately followed after PE, it showed no significant benefit, although PE is proposed widely to be an important component of frailty management [44]. The improvements observed in the intervention groups could be attributed to improved physical fitness and cognitive function and therefore reduced frailty, a geriatric syndrome including both physical and cognitive dimensions. On the contrary, video watching is less likely to bring cognitive benefits when compared with CCT, therefore it is reasonable that the control participants experienced fewer reductions in frailty levels. Alternatively, it could be that the baseline levels of frailty in the control participants were relatively low, therefore the improvements did not reach statistical significance. These findings have rarely been demonstrated in the literature. By contrast, a few previous trials using traditional cognitive training with/without combination of PE have proven beneficial in improving motor balance and gait speed [45,46] and reducing frailty levels [47]. It therefore seems that cognitive training is needed to reduce frailty levels. This finding provides new perspectives in understanding the effects of CCT in preventing frailty.

This study also confirmed the hypothesis that CCT combined with PE would yield a larger benefit in cognitive function as shown by global cognition, total learning ability and verbal memory ability compared with video watching combined with PE. The findings concur with the findings from several trials [31,32,33], which showed a significantly larger improvement in memory, and from a meta-analysis [48], which showed a significantly larger improvement in global cognition in the intervention group combining cognitive and physical training than the control group. While PE can increase the number of neurons [49,50,51] and para-hippocampal cerebral blood flow, both of which enhance neural efficiency and possibly cognition [52], CCT, instead of video watching, can potentially increase the number of surviving neurons [53], facilitating pattern separation [54], memory resolution [55] and timing and/or cognitive flexibility [56], which are important processes related to learning and memory. It is also likely that PE (including acute exercise) increases levels of cortisol that could facilitate learning and memory during CCT and hence provides additional cognitive benefits. For example, a single bout of aerobic exercise can facilitate learning mechanisms within visual and motor domains [57]. This is reminiscent of data from a recent study of middle-aged adults which shows that both aerobic and balance exercise were able to positively affect cognitive performance including reaction time, perceptual speed and executive control [58]. A meta-analysis also shows that a bout of exercise provided benefits in cognitive performance [59]. 

In addition to the potential effects of CCT preceded by PE, the different effects of the multi-domain and the two-domain CCT were examined in the present study. Previous studies have suggested that multi-domain cognitive training would have broader beneficial effects on cognition compared with single-domain cognitive training [60]. However, our findings indicated that, although the multi-domain CCT appears to confer a larger improvement in global cognition and executive functions, and that the two-domain CCT appears to confer a larger effect on memory, the changes did not reach statistical significance. This could be that the intensity/dosage of the CCT may not be sufficient to achieve significant between-group differences. Further study is needed to clarify whether multi-domain CCT is more effective in improving cognitive function than domain-specific CCT.

It has also been argued that the efficiency of cognitive training may be dependent on the cognitive ability of the participants. It may be that only participants who have maintained a high cognitive status profit from interventions. Alternatively, it may be that participants with lower cognitive status would benefit more from interventions. However, our findings indicated significant training effects on frailty, total learning ability, verbal memory ability and executive functions, independent of the baseline cognition of the participants.

The interventions were well-received, with all participants returning for follow-up assessments. However, there are some limitations of the study. Firstly, a CCT alone group and a passive control group were not included. Secondly, the study did not examine whether the observed benefits endure beyond the period of training. Thirdly, RCS may not be sensitive in detecting the changes in cognitive function. Fourthly, the relatively small sample size of the sub-groups according to RCS scores has limited the interpretation of our findings. Finally, the exclusion of participants with frailty/history of cognitive impairment/dementia, and mostly women, have limited the generalizability of our findings.

## 5. Conclusions

In conclusion, a combined intervention of CCT preceded by PE in community settings improves frailty status and cognitive function of community-dwelling older adults, which underscores the importance of multi-component interventions as possible strategies to reduce frailty levels and optimize cognitive function in older adults. Further studies should include older adults living with frailty and/or with MCI or dementia. A larger sample size with longer follow-up would also help to determine if the improvements in frailty status and cognitive function are maintained following completion of the intervention, or if the intervention needs to be continued to maintain these benefits.

## Figures and Tables

**Table 1:** 17 interactive touchscreen video games of the *Brainastic* computerized cognitive training.

Name of Video Game	Game Details	Cognitive Domain Focus
1. Forest of Memory	Remember the location of the cards with different symbols to train short-term memory	Memory
2. Catch the Star	Remember the order of the stars that change in color to train memory and logical thinking	Memory
3. Colored Light Bulbs	Remember the color of the light bulbs to train short-term memory	Memory
4. Master of Oriental Stitch	Remember the pattern and color of embroidery and recognize differences to train short-term memory	Memory
5. Conveyor Belt	Switch the conveyor sorter and collect items with a specific color to train multi-task handling capability	Attention
6. Spot the Difference	Find the unique insect to train attention and capability to filter information	Attention
7. Film Collector	Select the film with a specific color or pattern to train the immediate response to moving objects	Attention
8. Honey Haunters	Identify the correct number over a short period of time continuously to train attention and capability to filter information	Attention
9. Conquer the Ice	Look for the ice cubes with the answer to the calculation to train quick logical thinking	Executive functions
10. From Small to Big	Select labels with different presentations of values in ascending order to train quick logical thinking	Executive functions
11. Switch and Match	Pair the fish with same color but different pattern to train multiple condition handling capabilities	Executive functions
12. Piet Mondrian Mansion	Determine the consistency of the color of light and literal meaning of word of the lift to that on the wall to train quick conversion thinking	Flexibility
13. Color or Shape	Match the shape or color of moving signals to train quick conversion thinking	Flexibility
14. Save the Daruma	Match the pattern to the blocks on the left and right to train quick conversion thinking	Flexibility
15. Pairing Detective	Fit the totem pole to the empty slots on the wall to train sensitivity of visual space	Visuospatial
16. Fixing Pixels	Identify the dominant color of the grids on the TV screen to train the ability to distinguish colors	Visuospatial
17. Dance in the Rain	Select an umbrella with a specific color and shape to train sensitivity to multiple visual spaces	Visuospatial

**Table 2:** Baseline characteristics of the study population (*n* = 347).

	Mean ± SD/*n* (%)				
	Cognitive Intervention Group				
Variables	Total (*n* = 347)	Multi-Domain CCT + PE (*n* = 117)	Two-Domain CCT + PE (*n* = 116)	Video Watching + PE (Control) (*n* = 114)	*p* ^1^
Socio-demographics					
Age, years	64.2 ± 6.4	64.7 ± 7.3	64.0 ± 6.3	64.0 ± 5.3	0.659
Sex					0.056
Men	50 (14.4)	12 (10.3)	24 (20.7)	14 (12.3)	
Women	297 (85.6)	105 (89.7)	92 (79.3)	100 (87.7)	
Marital					0.977
Not married	114 (32.9)	38 (32.5)	39 (33.6)	37 (32.5)	
Married	233 (67.1)	79 (67.5)	77 (66.4)	77 (67.5)	
Educational level					0.103
Secondary or below	232 (66.9)	74 (63.2)	73 (62.9)	85 (74.6)	
Post-secondary or above	115 (33.1)	43 (36.8)	43 (37.1)	29 (25.4)	
Living arrangement					0.586
Living with others	298 (85.9)	103 (88.0)	100 (86.2)	95 (83.3)	
Living alone	49 (14.1)	14 (12.0)	16 (13.8)	19 (16.7)	
Frailty					
FRAIL, score	0.9 ± 0.7	0.9 ± 0.7	0.9 ± 0.7	0.7 ± 0.7	0.056
Cognitive function					
RCS, score	7.1 ± 1.6	7.0 ± 1.7	7.2 ± 1.4	7.2 ± 1.6	0.472
HKLLT-TL, score	27.7 ± 7.0	27.2 ± 6.9	27.5 ± 7.3	28.6 ± 6.5	0.237
HKLLT-DRT, score	9.4 ± 3.5	9.2 ± 3.1	9.1 ± 3.6	9.9 ± 3.6	0.155
FAB, score	14.9 ± 2.4	14.5 ± 2.4	15.6 ± 2.1	14.7 ± 2.6	0.001

CCT = computerized cognitive training; FAB = Frontal Assessment Battery; FRAIL = simple frailty questionnaire; HKLLT-TL/DRT = Hong Kong List Learning Test-Total Learning/Delay Recall Trial; PE = physical exercise; RCS = Rapid Cognitive Screen. Data are presented as mean ± standard deviation, or number (percentage) of each of the variables. ^1^ *p*-value of difference between the multi-domain CCT + PE, the two-domain CCT + PE and the video watching + PE (control) groups.

**Table 3:** Changes in outcome measures by intervention group (*n* = 347).

	Mean ± SD				Mean ± SD					Mean ± SD					
	Multi-Domain CCT + PE (*n* = 117)				Two-Domain CCT + PE (*n* = 116)					Video Watching + PE (Control) (*n* = 114)					
Variables	Pre	Post	Diff	*p* ^1^	Pre	Post	Diff	*p* ^1^	*p* ^2^	Pre	Post	Diff	*p* ^1^	*p* ^3^	*p* ^4^
FRAIL	0.9 ± 0.7	0.5 ± 0.7	−0.4 ± 1.0	<0.001	0.9 ± 0.7	0.5 ± 0.6	−0.4 ± 0.8	<0.001	0.794	0.7 ± 0.7	0.7 ± 0.8	−0.1 ± 0.9	0.319	0.011	0.012
RCS	7.0 ± 1.7	7.6 ± 1.5	0.6 ± 2.0	0.002	7.2 ± 1.4	7.7 ± 1.7	0.5 ± 1.8	0.004	0.700	7.2 ± 1.6	7.5 ± 1.4	0.3 ± 2.0	0.124	0.455	0.390
HKLLT-TL	27.2 ± 6.9	32.4 ± 6.6	5.2 ± 6.8	<0.001	27.5 ± 7.3	33.4 ± 7.1	6.0 ± 5.9	<0.001	0.375	28.6 ± 6.5	32.0 ± 7.3	3.4 ± 8.0	<0.001	0.014	0.015
HKLLT-DRT	9.2 ± 3.1	10.9 ± 3.4	1.7 ± 3.4	<0.001	9.1 ± 3.6	11.5 ± 3.4	2.4 ± 3.4	<0.001	0.133	9.9 ± 3.6	11.1 ± 3.5	1.2 ± 3.8	0.001	0.038	0.027
FAB^^^	14.5 ± 2.4	15.9 ± 2.3	1.4 ± 2.7	<0.001	15.6 ± 2.1	16.5 ± 1.8	0.9 ± 2.2	<0.001	0.162	14.7 ± 2.6	16.0 ± 2.1	1.3 ± 3.0	<0.001	0.345	0.371

CCT = computerized cognitive training; FAB = Frontal Assessment Battery; FRAIL = simple frailty questionnaire; HKLLT-TL/DRT = Hong Kong List Learning Test-Total Learning/Delay Recall Trial; PE = physical exercise; RCS = Rapid Cognitive Screen. Data are presented as mean ± standard deviation of the score of each scale. ^^^ Missing data: FAB, *n* = 1. ^1^ *p*-value of difference within group. ^2^ *p*-value of difference between the multi-domain CCT + PE and the two-domain CCT + PE groups. ^3^ *p*-value of difference between the multi-domain CCT + PE, the two-domain CCT + PE and the video watching + PE (control) groups. ^4^ *p*-value of difference between the multi-domain CCT + PE, the two-domain CCT + PE and the video watching + PE (control) groups adjusted for FAB score at baseline.

**Table 4:** Changes in outcome measures by intervention group and baseline cognition, RCS ≥ 8 (*n* = 135) and RCS < 8 (*n* = 212).

	Mean ± SD				Mean ± SD					Mean ± SD					
	Multi-Domain CCT + PE (*n* = 41)				Two-Domain CCT + PE (*n* = 47)					Video Watching + PE (Control) (*n* = 47)					
Variables	Pre	Post	Diff	*p* ^1^	Pre	Post	Diff	*p* ^1^	*p* ^2^	Pre	Post	Diff	*p* ^1^	*p* ^3^	*p* ^4^
RCS ≥ 8 (*n* = 135)															
FRAIL	0.9 ± 0.8	0.4 ± 0.7	−0.4 ± 1.0	0.015	0.9 ± 0.6	0.5 ± 0.7	−0.4 ± 0.8	0.005	0.794	0.7 ± 0.7	0.6 ± 0.7	−0.1 ± 0.8	0.390	0.230	0.228
HKLLT-TL	29.5 ± 6.6	33.9 ± 6.1	4.4 ± 6.6	<0.001	28.8 ± 7.1	35.2 ± 6.3	6.3 ± 5.9	<0.001	0.143	30.1 ± 6.0	33.7 ± 7.2	3.6 ± 7.9	0.003	0.137	0.161
HKLLT-DRT	10.4 ± 2.8	11.7 ± 3.1	1.3 ± 3.4	0.019	9.2 ± 3.7	12.4 ± 2.3	3.1 ± 3.1	<0.001	0.009	10.7 ± 3.3	11.5 ± 3.6	0.7 ± 4.0	0.206	0.004	0.004
FAB	15.2 ± 2.0	16.4 ± 1.7	1.3 ± 2.5	0.003	15.7 ± 2.1	16.5 ± 1.8	0.8 ± 2.2	0.019	0.343	15.3 ± 2.4	16.2 ± 2.1	0.9 ± 2.8	0.031	0.647	0.762
RCS < 8 (*n* = 212)															
FRAIL	1.0 ± 0.7	0.6 ± 0.7	−0.4 ± 1.0	<0.001	0.9 ± 0.7	0.5 ± 0.6	−0.4 ± 0.8	<0.001	0.916	0.8 ± 0.7	0.7 ± 0.8	−0.1 ± 1.0	0.545	0.047	0.047
HKLLT-TL	25.9 ± 6.8	31.6 ± 6.7	5.7 ± 6.9	<0.001	26.5 ± 7.4	32.3 ± 7.3	5.7 ± 6.0	<0.001	0.979	27.6 ± 6.8	30.8 ± 7.2	3.2 ± 8.1	0.002	0.056	0.054
HKLLT-DRT	8.6 ± 3.1	10.5 ± 3.5	1.9 ± 3.5	<0.001	9.0 ± 3.6	10.8 ± 3.9	1.9 ± 3.6	<0.001	0.930	9.3 ± 3.7	10.8 ± 3.4	1.5 ± 3.7	0.002	0.728	0.671
FAB ^^^	14.1 ± 2.5	15.6 ± 2.5	1.4 ± 2.9	<0.001	15.5 ± 2.2	16.5 ± 1.8	1.0 ± 2.2	<0.001	0.314	14.2 ± 2.7	15.9 ± 2.1	1.7 ± 3.2	<0.001	0.365	0.264

CCT = computerized cognitive training; FAB = Frontal Assessment Battery; FRAIL = simple frailty questionnaire; HKLLT-TL/DRT = Hong Kong List Learning Test-Total Learning/Delay Recall Trial; PE = physical exercise; RCS = Rapid Cognitive Screen. Data are presented as mean ± standard deviation of the score of each scale. ^^^ Missing data: RCS < 8 (*n* = 212), FAB, *n* = 1. ^1^ *p*-value of difference within group. ^2^ *p*-value of difference between the multi-domain CCT + PE and the two-domain CCT + PE groups. ^3^ *p*-value of difference between the multi-domain CCT + PE, the two-domain CCT + PE and the video watching + PE (control) groups. ^4^ *p*-value of difference between the multi-domain CCT + PE, the two-domain CCT + PE and the video watching + PE (control) groups adjusted for FAB score at baseline.

## Data Availability

Data sharing is not applicable to this article.
